# A Study on Photostability of Amphetamines and Ketamine in Hair Irradiated under Artificial Sunlight

**DOI:** 10.3390/brainsci8060096

**Published:** 2018-05-28

**Authors:** Giorgia Miolo, Marianna Tucci, Luca Menilli, Giulia Stocchero, Susanna Vogliardi, Salvatore Scrivano, Massimo Montisci, Donata Favretto

**Affiliations:** 1 Department of Pharmaceutical and Pharmacological Sciences, University of Padova, 35131 Padova, Italy; luca.menilli@phd.unipd.it; 2 Legal Medicine and Toxicology, University Hospital of Padova, 35121 Padova, Italy; marianna.tucci@gmail.com (M.T.); giulia.stocchero@aopd.veneto.it (G.S.); susanna.vogliardi@unipd.it (S.V.); massimo.montisci@unipd.it (M.M.); 3 School of Specialization in Legal Medicine, University Hospital of Padova, 35121 Padova, Italy; salvatore.scrivano@studenti.unipd.it

**Keywords:** hair, solar light, photodegradation, amphetamines, MDMA, ketamine

## Abstract

Drugs incorporated into hair are exposed to the environment, and cosmetic and chemical treatments, with possible decreases in their content. Knowledge concerning the effect of sunlight on drug content in hair can be helpful to forensic toxicologists, in particular, when investigating drug concentrations above or below pre-determined cut-offs. Twenty authentic positive hair samples were selected which had previously tested positive for amphetamines and/or ketamine. Washed hair were divided into two identical strands, with the former exposed at 765 W/m^2^ (300–800 nm spectrum of irradiance) for 48 h in a solar simulator, and the latter kept in the dark. Hair samples were extracted and analyzed by liquid chromatography high-resolution mass spectrometry detection. The percentage of photodegradation was calculated for each analyte (i.e., amphetamine, methamphetamine, methylendioxyamphetamine, ketamine, and norketamine). In parallel, photodegradation processes of standard molecules dissolved in aqueous and organic solutions were studied. In 20 hair samples positive for the targeted analytes, exposure to artificial sunlight induced an appreciable decrease in drug concentrations. The concentration ranges in the non-irradiated hair samples were 0.01–24 ng/mg, and 65% of samples exhibited a decrease in post-irradiation samples, with reduction from 3% to 100%. When more drugs were present in the same hair sample (i.e., MDMA and ketamine) the degradation yields were compound dependent. A degradation product induced by irradiation of ketamine in aqueous and methanol solutions was identified; it was also found to be present in a true positive hair sample after irradiation. Ketamine, amphetamines, and their metabolites incorporated in the hair of drug users undergo degradation when irradiated by artificial sunlight. Only for ketamine was a photoproduct identified in irradiated standard solutions and in true positive irradiated hair. When decisional cut-offs are applied to hair analysis, photodegradation must be taken into account since sunlight may produce false negative results. Moreover, new markers could be investigated as evidence of illicit drug use.

## 1. Introduction

The main advantage of hair as a testing matrix is the ability to provide information relating to historical drug exposure. Hair analysis has many applications within forensic (e.g., drug-related deaths, drug-facilitated crimes (DFCs), child protection) and clinical toxicology (e.g., drug rehabilitation programs, workplace drug testing) [1,2,3,4].

The stability of drugs in hair, however, is affected by exposure to sunlight and weathering, cosmetic chemical treatments (i.e., oxidative dyeing, bleaching, or permanent wave), and physical damage [5,6,7]. In particular, exposure to sunlight and/or artificial light for many hours per day can induce photodegradation (i.e., photolysis) of licit/illicit drugs through the formation of free radicals (produced by the drug itself or formed by eumelanin and pheomelanin, and their oxidative products oxyeumelanin, and oxypheomelanin) or photosensitization reactions by intermolecular energy transfer. [8]

Previous studies on this matter have been published by Skopp et al. [9] in which cannabinoids detected in hair and affected by solar radiation were shown to reduce in concentration. More recently, Favretto et al. [10] evaluated the effect of light exposure on methadone, cocaine, and heroin metabolites in hair.

In order to better understand the role and underlying mechanisms of solar light exposure on decreasing concentrations of drugs in hair, and following our previous [10] photodegradation studies on UVA- and UVB-induced changes, the aim of the present work was to evaluate the photodegradation of several common stimulant drugs (i.e., amphetamines and ketamine (KET)) in true positive hair samples exposed to the whole spectrum of sunlight in a solar simulator. The use of a solar simulator, including visible light (400–800 nm) and part of UV radiation from 400 nm to 3 µnm (UVA = 400–315 nm, UVB = 315–280 nm), mimics exposure to environmental sunlight.

Amphetamine-based drugs are illegal synthetic stimulants that share a common structural backbone. The four amphetamines considered in the present work are the most used in Europe: amphetamine (AMF), methamphetamine (MA), 3,4-methylenedioxymethamphetamine (MDMA), and 3,4-methylenedioxyamphetamine (MDA).

Amphetamine can be metabolized along two pathways: either by hydroxylation of the aromatic ring to 4-hydroxyamphetamine or by deamination of the side chain to benzyl methyl ketone, which can then be degraded to benzoic acid. Methamphetamine is metabolized by cytochrome P450 (CYP), mainly by the CYP2D and CYP3A subfamilies, leading to the production of 4-hydroxyamphetamine and AMF. The half-life of MA is about 10 h, and 35–45% of a dose is excreted unchanged in the urine over a period of several days. Other metabolites, such as 4-hydroxymethamphetamine, norephedrine, and 4-hydroxynorephedrine, can also be found in urine in substantial quantities [11,12,13].

In addition, MDMA (i.e., 3,4-methylenedioxymethamphetamine, ecstasy) is a widely abused psychostimulant drug that acts as a powerful releaser and/or reuptake inhibitor of serotonin (5-HT), dopamine (DA), and norepinephrine (NE). Its metabolism depends on the following main metabolic pathways: (1) *O*-demethylenation followed by catechol-*O*-methyltransferase (COMT) methylation and/or glucuronide/sulfate conjugation; and (2) *N*-dealkylation, deamination, and oxidation. MDMA *N*-demethylation gives rise to 3,4-methylenedioxyamphetamine (MDA). The elimination half-life of MDMA is about 8–9 h, lower than those reported for MA (10–12 h) or AMF (12–15 h) [14,15].

Ketamine is a dissociative anesthetic drug that functions as an antagonist of the *N*-methyl-d-aspartate receptor and enhances the antinociceptive effects of conventional opioid analgesia, binding to μ opioid and σ receptors. Ketamine is increasingly misused as a recreational and “club drug” because of its hallucinogenic and stimulant effects, and also as a “date-rape” drug (to facilitate sexual assault). Ketamine is metabolized in the liver by the P450 system, and CYP3A4 is the main enzyme responsible for transforming *N*-demethylation into norketamine (NKET), 4-hydroxy-ketamine, and 6-hydroxy-ketamine. The elimination half-life is about 2 h, and the predominant metabolite of urinary excretion over a 72 h period is dehydronorketamine (DHNK) (16%), along with conjugates of hydroxylated ketamine metabolites [16,17].

The consumption of alcohol and/or drugs is associated with an increased risk of being the victim of a sexual assault. A retrospective case series in London on 1014 cases of claimed drug-facilitated sexual assaults (DFSAs) showed that in 34% of samples (blood and/or urine), an illicit drug (with or without alcohol) was found, of which 10.8% contained cocaine, 4.6% “ecstasy” (MDMA), and 2.3% AMF [18,19]. In DFC cases, alleged victims are often unconscious leading up to the assault due to the amnesiac effects of the drug(s) administered, thus a considerable amount of time may be spent before the victim reports the incident. Consequently, hair analysis is of primary importance compared to blood and urine analysis, particularly when the occasional consumption is sufficiently high; however, exposure to the environment may affect the stability of drugs in the keratin matrix.

For these reasons, reliable interpretation of the analytical results is fundamental for a correct interpretation of a positive or negative result, and general knowledge of the photostability of drug analytes in the biological matrix must be considered. However, no data have been published until now on the effect of light on KET and amphetamines, and their respective metabolites in the keratin matrix; only morphine and 6-monoacetylmorphine (6-MAM) photodegradation have been studied by UVA and UVB irradiation [20].

In the present paper, levels of AMF, MA, MDMA, MDA, KET, and NKET were determined by means of liquid chromatography high-resolution mass spectrometry (HPLC-HRMS) [21,22] in authentic hair samples from drugs users before and after irradiation under the whole spectrum of sunlight in a solar simulator.

Although the photodegradation of a molecule not only depends on the wavelength used, but also on its physical state (solid or liquid) and the environment in which it is irradiated (i.e., solvent, polarity, pH, and the presence of salts, oxygen, and other compounds in the sample), we tested the same analytes in a pure methanol and water solution irradiated under the whole spectrum of sunlight to gather general information on their behavior and to study their kinetics of photodegradation.

## 2. Experiments

### 2.1. Chemicals

Amphetamine, MA, MDMA, MDA, KET, and NKET were purchased from LGC Promochem Cerilliant (Teddington, Middlesex, UK) as pure solutions in methanol at 1.0  mg/mL. The internal standards (IS) MA-D5, AMF-D5, MDMA-D5, MDA-D5, and KET-D3 were methanol solutions also from LGC Promochem at 1.0  mg/mL.

Methanol, dichloromethane, and acetonitrile (Merck, Darmstadt, Germany) were high-performance liquid chromatography (HPLC) grade. Trifluoroacetic acid (TFA) was from Sigma (Sigma-Aldrich, Milan, Italy). High-performance liquid chromatography water was prepared using a Milli-Q Plus (Millipore, Molsheim, France) system. All other reagents were from Sigma-Aldrich (St Louis, MO, USA).

### 2.2. Hair Samples

Authentic positive hairs were selected from samples at the laboratory that had previously tested positive for amphetamines and/or KET. Hair samples were collected with scissors from the posterior vertex and cut as close to the scalp as possible, wrapped in aluminum foil, and kept at room temperature until analysis. As recorded in donor interviews, all samples had not been submitted to previous cosmetic or chemical treatments.

### 2.3. Irradiation Procedure

Irradiation was performed in a Suntest CPS+ (Atlas, Linsengericht, Germany) equipped with a 1.8 kW xenon lamp and a glass filter (cut-off 310 nm) according to Option 1 of ICH Guideline Q1B (European Medicines Agency, London, UK, ICH Topic Q1B–Photostability testing of new active substances and medical products, 1998). The dark samples were maintained at solar box temperature during irradiation.

### 2.4. Photolysis Experiments in Solution (In Vitro Study)

Solutions of the compounds at concentrations ranging from 10^−5^ to 10^−4^ M in methanol and water were irradiated in the Suntest CPS+ with times increasing from 1 h up to 3 h.

Photolysis was evaluated using Cary 50 UV-Vis spectrophotometer (Varian, Milan, Italy) analyzing the change in the original spectrum upon irradiation, as already described [20], and by high-resolution mass spectrometry (HRMS). At selected UV doses, the solutions were diluted to 10^−6^ M in methanol and water, and analyzed by direct injection HRMS to measure the photodegradation of the analytes by recording the decrease of the ion signal of protonated molecules obtained by electrospray ionization (ESI) of solutions kept in the dark. The results are the mean of at least three experiments.

### 2.5. Photolysis Experiments in Hair Samples (In Vivo Study)

Hairs 5–7 cm long were divided into two approximately identical strands: the former was put between two 5 × 5 cm optical glasses and exposed at 765 W/m^2^ (spectrum of irradiance: 310–800 nm) for 48 h in the solar simulator to an endpoint corresponding to two months exposure under the sunlight, and the latter was kept as a dark control in the same chamber of irradiation covered with an aluminum foil.

### 2.6. Hair Sample Preparation and Extraction

Hair samples were decontaminated with 3 mL of water and 3 mL of methanol. Pulverization was applied by the automatic homogenizer Precellys^®^ Evolution (Bertin Technologies, Genoa, Italy) at a speed of 6000 RPM, cycle 9 × 30 s, pause 30 s. Solid phase extraction (SPE) was performed by Oasis MCX (Waters, Milford, MA, USA) cartridges. Sample preparation consisted of the addition of 2 ng/mg of IS and 3 mL of a methanol/TFA (90:10, *v*/*v*) solution to 25 mg powdered hair samples, ultrasonication for 1 h, and incubation overnight at 45 °C. After centrifugation, the methanolic solutions were dried under a stream of nitrogen at 40 °C. The residues were reconstituted in 3 mL of 0.1 M phosphate buffer pH 6 and subjected to SPE. After, cartridges of the sample conditioned with 3 mL methanol and 3 mL 0.1 M phosphate buffer pH 6 were loaded. Cleanup was accomplished by sequential washes with 3 mL of water, 3 mL of 0.1 N hydrochloric acid, and 3 mL of methanol. Cartridges were dried for 10 min under vacuum before elution with 2 mL of dichloromethane: 2-propanol (80:20, *v*/*v*) 2% ammonium hydroxide. Eluates were evaporated to dryness with nitrogen at 40 °C, reconstituted in 200 µL of water (0.1% formic acid)/acetonitrile 9:1 mixture, and 25 µL were injected into the LC-HRMS system.

### 2.7. High-Resolution Mass Spectrometry (HRMS)

All measurements were performed on an LTQ-Orbitrap (Thermo Fisher Scientific, Bremen, Germany) high-accuracy, high-resolution mass spectrometer operating in positive ESI mode and equipped with a Surveyor MS Pump.

### 2.8. Electrospray Ionization HRMS

For the analysis of pure standard solutions, direct injection analysis was performed with a syringe pump delivering solutions at 10 μL/min directly into the ESI source. The positive ion ESI parameters were as follows: capillary voltage 10  V, sheath gas flow rate 20 (arbitrary units, a.u.), auxiliary gas (N_2_) flow rate 5 (a.u.), sweep gas flow rate 5 (a.u.), and capillary temperature 275  °C. Profile full-scan mass spectra were acquired in the Orbitrap in the *m*/*z* range 120–700 with a target mass resolution of 100,000 (FWHM as defined at 400 *m*/*z*) and a scan time of 0.65  s.

### 2.9. High-Performance Liquid Chromatography HRMS

For the determination of drug concentrations in hair and in standard solutions, 25 μL of solutions or extracts were injected into an Atlantis T3 (150 × 1.0  mm, 3  µm) column (Waters Corporation, Milford, MA, USA). High-performance liquid chromatography separation was achieved by gradient elution at a constant flow rate of 300  μL/min. High-performance liquid chromatography conditions: A (water, 0.1% formic acid) and B (methanol, 0.1% HCOOH); initial conditions 10% B, linear gradient to 25% B in 4 min, 25% B hold from 4 min to 7 min, then ramped to 40% B in 5 min, to 60% B in 4 min and to 90% B in 2 min; 90% B hold from 18 min to 26 min. The column temperature was 40  °C. Mass spectrometry conditions were: positive ion ESI; capillary voltage 10  V, sheath gas flow rate 50 (arbitrary units, a.u.), auxiliary gas (N_2_) flow rate 5 (a.u.), sweep gas flow rate 5 (a.u.), and capillary temperature 275  °C. Profile full-scan mass spectra were acquired in the Orbitrap in the *m*/*z* range 120–700 with a target mass resolution of 60,000 (FWHM as defined at 400 *m*/*z*) and a scan time of 0.45  s. Detection of the analytes and the IS was based on retention time, accurate mass measurements of MH^+^ ions, and correspondence of the observed isotopic pattern to the calculated one. Drug concentrations were determined from peak area ratios of analyte to its IS compared to calibrator curves of peak area ratios to concentrations. The method was fully validated exhibiting a linear range from 0.1 ng/mg to 50 ng/mg (determined from regression with 1/x^2^ weighting utilizing six calibration points), lower limits of quantification of 0.01  ng/mg and limits of detection of 0.005  ng/mg for all the target analytes, intra-day imprecision, inaccuracy always lower than 19% and 20%, and inter-day imprecision and inaccuracy always lower than 21% and 22%, respectively. Extraction efficiency was determined in the range of 85–100% for the different substances.

## 3. Results

### 3.1. Photodegradation in Methanol and Water Solutions Exposed to Suntest CPS+

Preliminary, stock methanol, and water solutions for all the analytes diluted to 10^−4^ M were irradiated inside the photostability test chamber from 1 h to 3 h (each hour corresponding to about 70 J/cm^2^), and their absorption spectra before and after irradiation were recorded (Supplementary Figures S1A1–S1F2).

Amphetamine dissolved in methanol was characterized by the only band at around 205–210 nm, mainly corresponding to the solvent, thus remaining unmodified in all the irradiated solutions (see Supplementary Figure S1A1). However, a shift to 205 nm and a small increase in spectral intensity were detected by increasing irradiation. When irradiated in water, AMF was characterized by two bands (around 220 nm and 240 nm) decreasing as irradiation dose increased (see Supplementary Figure S1A2).

Methamphetamine, both in methanol and water (Figures S1B1 and B2, respectively), presented curves similar to AMF, with the unique difference being the absorption at 205 nm in water which increased under irradiation.

In addition, MDA in methanol (Figure S1C1) was characterized by three bands at 208 nm, 235 nm, and 285 nm. The irradiated solutions presented similar curves with increasing absorption and a small blue shift at 230 nm and 280 nm. Similar spectra were obtained for MDA in water (Figure S1C2), with two bands at 230 nm and 285 nm increasing in relation to irradiation time.

Furthermore, MDMA in methanol showed two bands centered at 235 nm and at 285 nm, indicating a small absorption increase and a small blue shift (Figure S1D1). A new band of absorption (300–340 nm) appeared which increased upon irradiation. In water, MDMA presented the same absorption band with a higher increase at 235 nm than at 285 nm, and the appearance of a band at 320 nm (Figure S1D2).

Ketamine in methanol was characterized by a single absorption band (205–230 nm). When irradiated, this band slightly increased and a shoulder (230–270 nm) appeared, as evidenced in Supplementary Figure S1E. On the contrary, in water, KET did not change its absorption spectrum upon irradiation (Figure S1E2). Absorption spectrum of NKET in methanol increased upon irradiation at 210–220 nm and in the range of 240–250 nm (Figure S1F1). In water, NKET showed a similar behavior upon irradiation for the band 240–250 nm, with slight changes (Figure S1F2).

### 3.2. Identification of New Photoproducts

**Figure 1:**
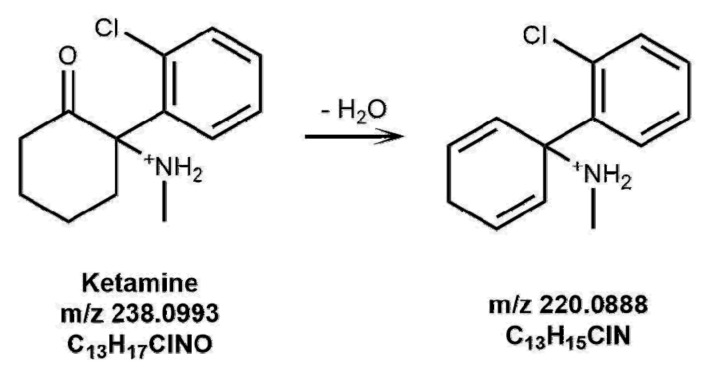
Proposed structures of ketamine (KET) photoproducts formed in a water solution upon irradiation for 3 h.

**Figure 2:**
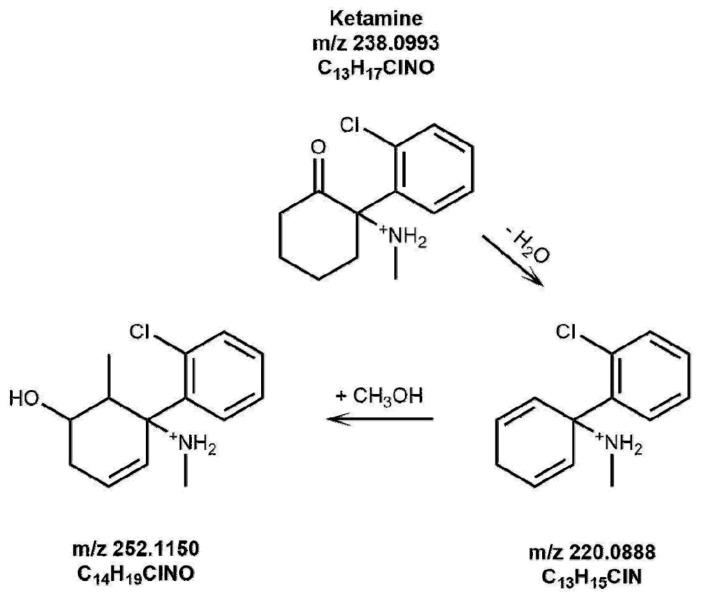
Proposed structures of KET photoproducts formed in a methanol solution upon irradiation for 3 h.

All the irradiated solutions in the solar simulator, including the control solutions kept in the dark, were diluted to 10^−5^ M either in methanol or water, and analyzed by direct infusion HRMS, with the aim of identifying the compounds eventually formed upon irradiation. Interestingly, for KET and NKET only, some new species were observed in irradiated solutions. In HRMS spectrum of KET irradiated in water solution, its [M+H]^+^ ion at 238.0993 *m*/*z* (C_13_H_17_ClNO) was accompanied by a new ionic species at 220.0888 *m*/*z* (C_13_H_15_ClN), corresponding to the loss of H_2_O. In methanol, two species were observed at 220.0888 *m*/*z* and at 252.0786 *m*/*z* (C_13_H_15_ClNO_2_), with the latter corresponding to the loss of H_2_ and to the photo-addition of one oxygen atom. The proposed structures of KET photoproducts are shown in Figure 1 and Figure 2. It must be highlighted that the ionic species identified were new protonated molecules present in solution, and they are not fragment ions produced by collisional experiments on precursor ions.

**Figure 3:**
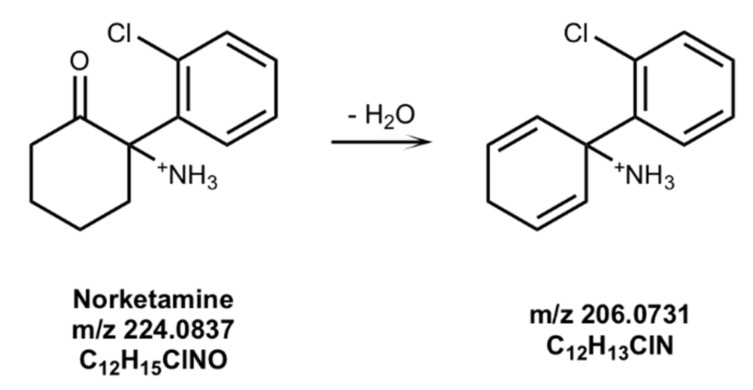
Proposed structures of norketamine (NKET) photoproducts formed in a methanol solution upon irradiation for 3 h.

In HRMS spectrum of the methanol solution of NKET irradiated in the solar simulator, [M+H]^+^ ions at 224.0837 *m*/*z* (C_12_H_15_ClNO) were accompanied by a new species at 206.0731 *m*/*z* (C_12_H_13_ClN), corresponding to the loss of H_2_O. The proposed structure of NKET photoproducts formed in methanol is shown in Figure 3 analogously to KET. However, differently from KET, no photoproduct was detected in HRMS spectrum of NKET irradiated in water solution.

**Figure 4:**
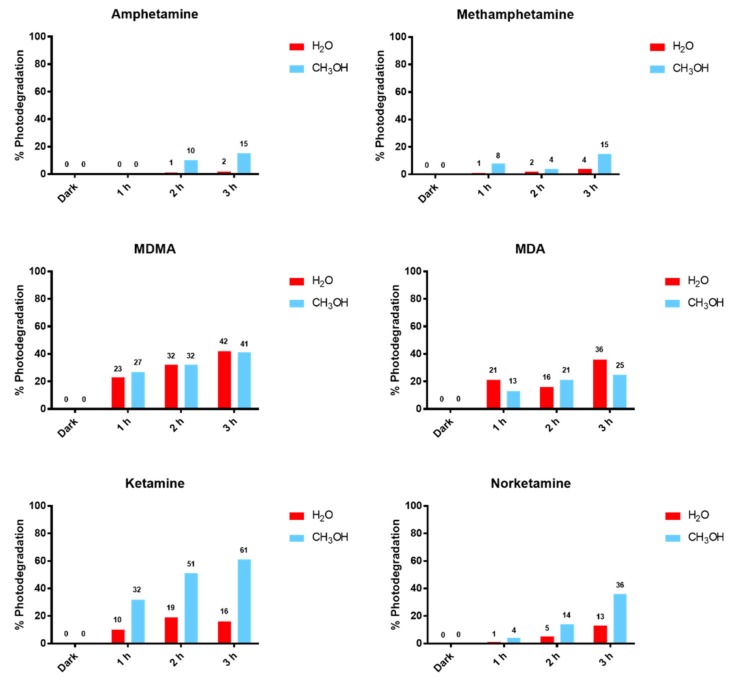
Relative degradation of target drugs in methanol and water solutions with increasing time of irradiation in the solar simulator (% photodegradation calculated using the peak area of protonated molecules in solutions kept in the dark as controls for 0% degradation).

Vice versa, for all the other analytes (i.e., AMF, MA, and MDMA), both in water and methanol solutions, no photoproducts were evidenced by direct HRMS analysis. To avoid ionization suppression phenomena that could occur in a mixture and shield the presence of less abundant species, irradiated solutions were also analyzed by HPLC-HRMS. The ratios of peak areas observed for samples upon irradiation vs those analogous samples kept in the dark were calculated as “percent degradation”. In Figure 4, the yields of photodegradation obtained for all the analytes after 1 h, 2 h, and 3 h of irradiation are reported.

As may be observed, AMF and MA in water show the highest photostability: the photodegradation was 1% and 4%, respectively, while in methanol it increased up to 15%. In addition, MDMA and MDA in methanol and water both presented similar photodegradation with a linear relationship with irradiation time: MDMA from 23% to 42% and MDA from 13% to 36%. For KET and NKET, the photodegradation yield was significantly higher in methanol (KET 61%; NKET 36%) than in water (KET 16%; NKET 13%) after 3 h.

### 3.3. Photodegradation in Hair Exposed to Solar Box

**Figure 5:**
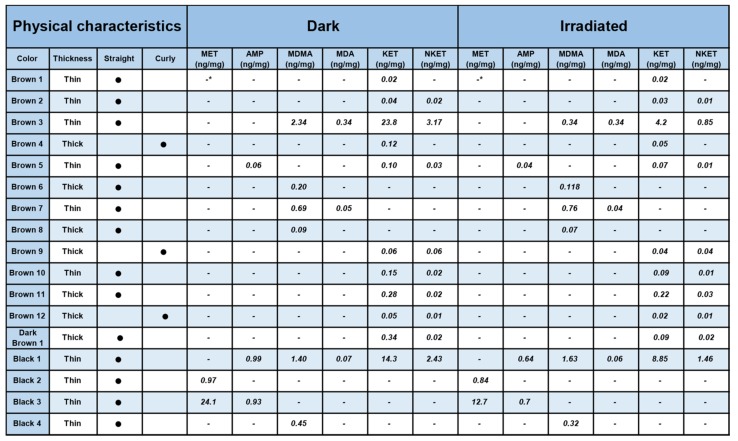
Drug concentrations in hair samples with their physical characteristics calculated by high-performance liquid chromatography high-resolution mass spectrometry (HPLC-HRMS) in both aliquots kept in the dark or irradiated in the solar simulator. -: absent at limit of detection (LOD, 0.005 ng/mg); (−) observed % increase.

The main goal of the study was to observe the photo-induced degradation of drugs in hair. In Figure 5, the concentrations of each drug for all seventeen hair samples irradiated in the solar simulator or kept in the dark (control) are presented.

**Figure 6:**
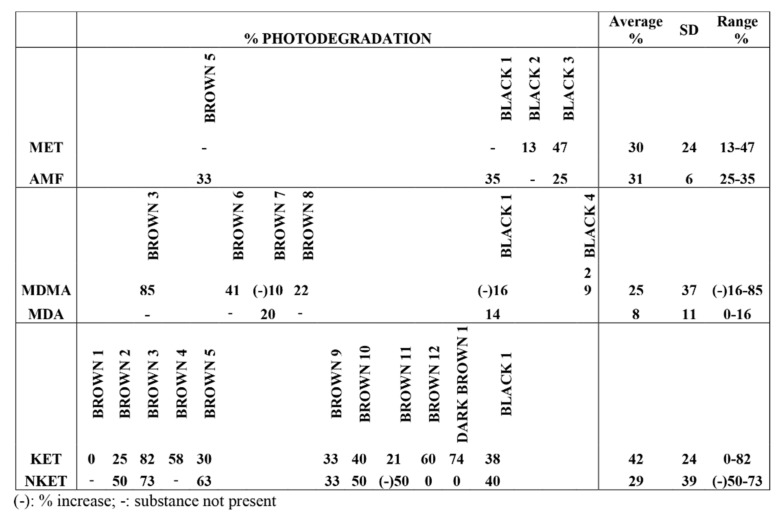
Percentage of photodegradation, average, standard deviation, and range of drugs in hair samples irradiated at 765 W/m^2^ in the solar simulator.

In Figure 6, the percentages of photodegradation are reported. Calculations were made using the concentrations of samples kept in the dark as control:

% photodegradation  =  (drug conc._dark_ − drug conc._irradiatied_)/drug conc._dark_ × 100.

**Figure 7:**
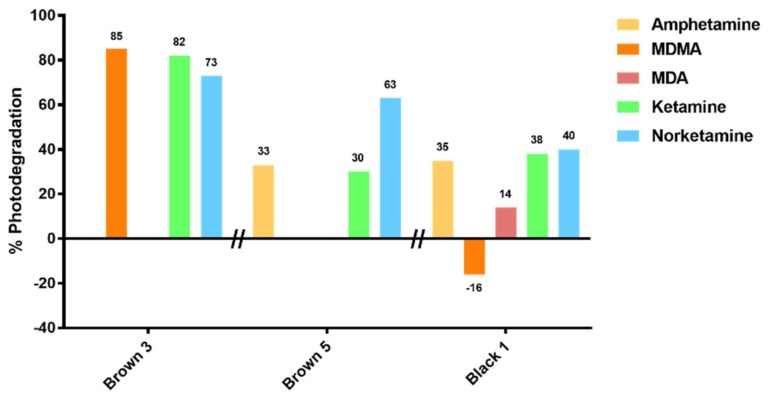
Percentage of photodegradation of amphetamine (AMF), KET, and NKET in three different hair samples. In these three samples, photodegradation seems to depend on the hair owner.

When comparing results from three poly-drug abusers, photodegradation of all the analytes was generally obtained, with the highest comparable photodegradation yields observed in sample Brown 3 (see Figure 7).

## 4. Discussion

From our results, a clearly different behavior of KET and NKET was evidenced when compared to all the other drugs and metabolites under study. Indeed, these drugs seem photo-unstable, both in solution and in hair matrix. In particular, for the experiments in solution, KET and NKET photo-induced products were identified; in hair samples their degradation was higher (average 42% and 29%) than all the other compounds (average 8–30%).

No color effect seems to be present, although no fair hair samples were present in this study, and the number of samples was limited. It is well known that the color of hair depends on the relative amount of pheomelanin (red) and eumelanin (black), the first defense against UV in human hair and skin [23,24,25,26]. Generally, a part of the light is absorbed by the hair matrix itself without any photochemical effect. In dark hair, the eumelanin can protect the drugs/metabolites with a higher degree than in fair hair. However, melanin may also react with oxygen under irradiation, producing reactive species, such as superoxide anion, that can induce photolysis of melanin itself [27], thus weakening the photoprotective effect of the pigment. In this context, no clear-cut interpretation of the role of hair color can be made.

Regarding MA and AMF, on the basis of the in vitro experiments, they were expected to photodegrade less readily than KET and NKET. Unexpectedly, the experiments in hair revealed an average degradation of 30% and 31% for MA and AMF, respectively, with a range of 13–47% and 25–35%.

**Figure 8:**
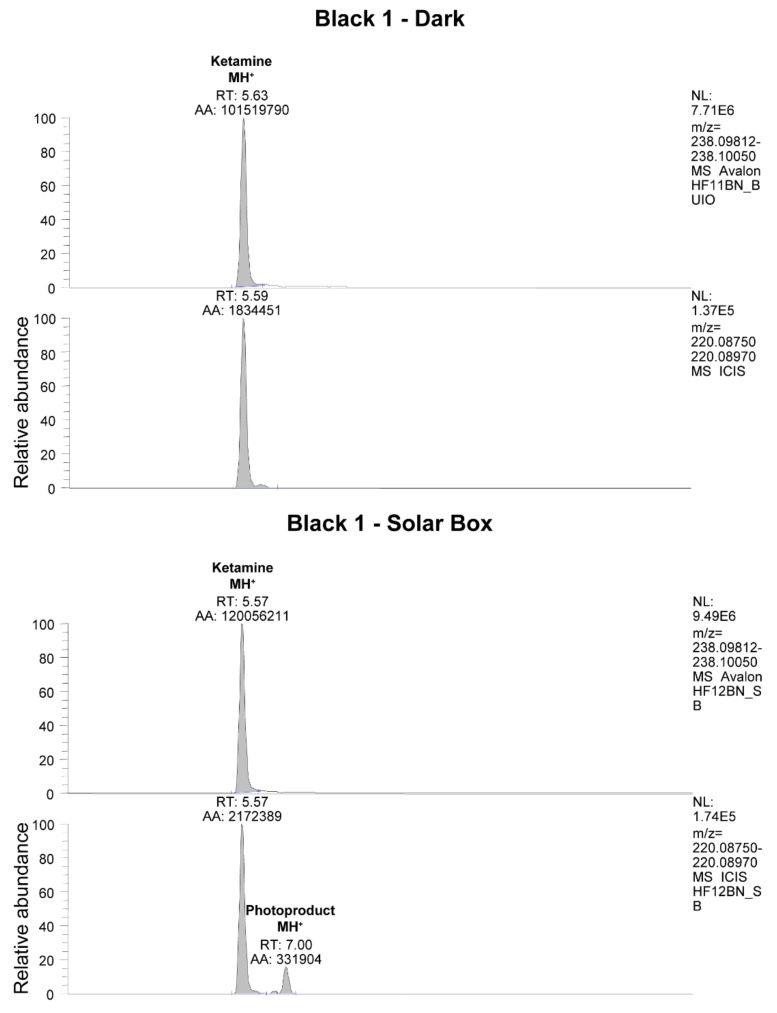
LC-HRMS analysis of hair sample Black 1 taken in the dark and after irradiation in the solar simulator. In the irradiated sample, the presence of a KET photoproduct with 220.08750 *m*/*z* is evident.

The presence of photoproducts was also investigated in irradiated hair samples. In one sample, the species at 220.08750 *m*/*z*, corresponding to the photoproduct of KET shown in Figure 1, could be identified by LC-HRMS as evidenced in Figure 8.

The importance of this finding must be highlighted, since no previous study has identified stable photoproducts in hair for KET, nor for any other substance previously investigated (i.e., cannabinoids, cocaine, opiates, methadone [9,10,20,21]).

The “apparent production” of NKET upon irradiation of sample Brown 11 was at first sight surprising, but could be reasonably related to the physical decomposition of the matrix, leaving KET more labile during the irradiation experiment and favoring its transformation to the metabolite. Furthermore, the increase of MDMA in sample Black 1 and Brown 7 upon irradiation can be rationalized (as already demonstrated in previous studies) by a greater lability of the keratin matrix with consequent greater yield during the extraction of the analytes in the liquid acid phase with access to deeper layers of the hair structure.

## 5. Conclusions

Amphetamine, MA MDA, MDMA, KET, and NKET incorporated in hair undergo degradation when irradiated by artificial sunlight, suggesting that they can suffer photodegradation under natural sunlight. With this work, for the first time, the presence of a photoproduct of KET was evidenced in one true positive sample.

Since the detection of drugs in hair is often used as evidence of illegal acts with consequences on the freedom of persons, the UV-Vis effects on the integrity of the drugs and their metabolites should be considered when single administration needs to be evidenced. When decisional cut-offs are applied to hair analysis (e.g., for granting a driving license, a job, or a child custody), it must be taken into account that hair exposed to sunlight may produce false negative results and lead to misjudgment. When possible, the detection of photoproducts of a drug under investigation can be a key factor in a case management.

## Supplementary Materials

The following are available online at http://www.mdpi.com/2076-3425/8/6/96/s1, Fig S1A1/A2. UV spectrum of AMF (10-4 M) in methanol/water, by increasing exposure time in solar simulator; Fig S1B1/B2. UV spectrum of MA (10-4 M) in methanol/water, by increasing exposure time in solar simulator; Fig S1C1/C2. UV spectrum of MDA (10-4 M) in methanol/water, by increasing exposure time in solar simulator; Fig S1D1/D2. UV spectrum of MDMA (10-4 M) in methanol/water, by increasing exposure time in solar simulator; Fig S1E1/E2. UV spectrum of KET (10-4 M) in methanol/water by increasing exposure time in solar simulator; Fig S1F1/F2. UV spectrum of NKET (10-4 M) in methanol/water, by increasing exposure time in solar simulator.
