# Supplementary material for: A Study on Photostability of Amphetamines and Ketamine in Hair Irradiated under Artificial Sunlight
Source: Brain Sci. 2018 May 28;8(6):96. doi: 10.3390/brainsci8060096 (PMC6025081; doi:10.3390/brainsci8060096)
Supplement: Supplementary file 1 [file brainsci-08-00096-s001.pdf]

Supplementary figures (S1A1-S1F2)

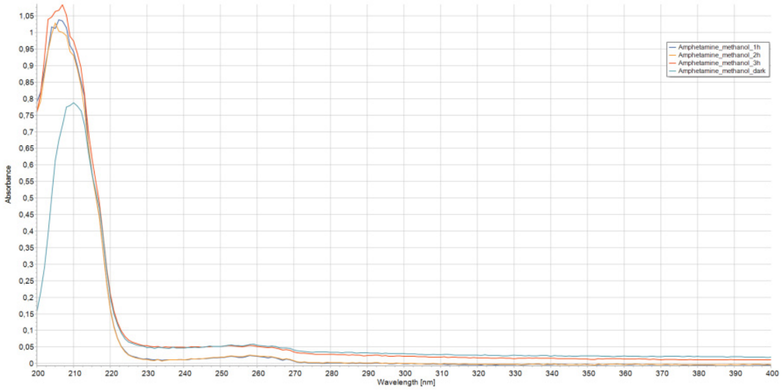

Figure S1A1

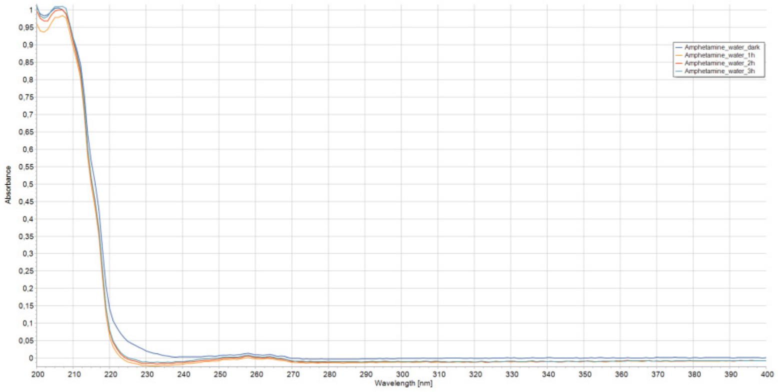

Figure S1A2

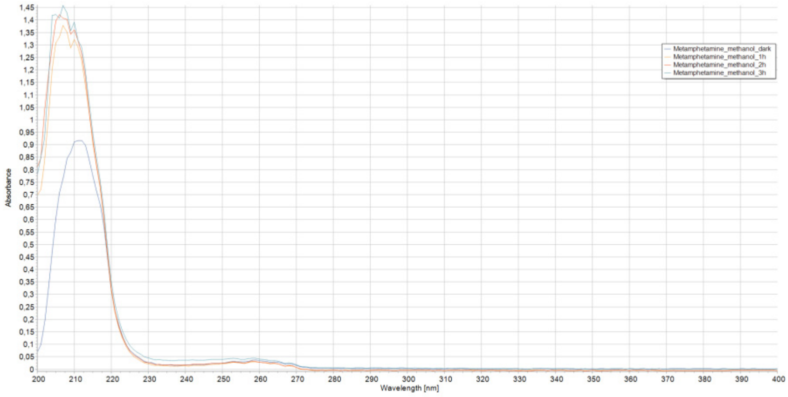

Figure S1B1

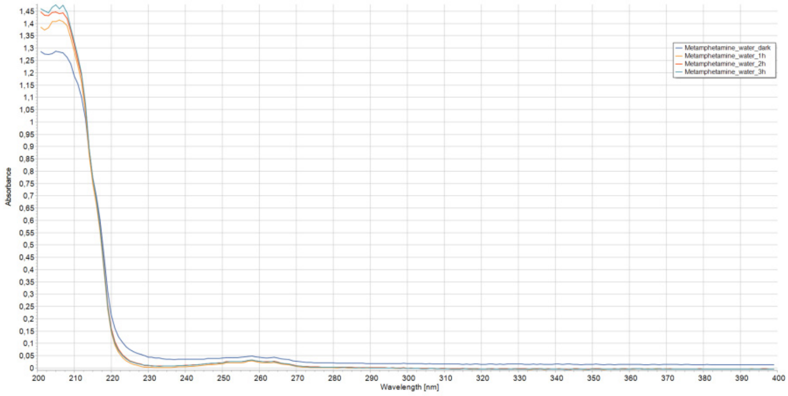

Figure S1B2

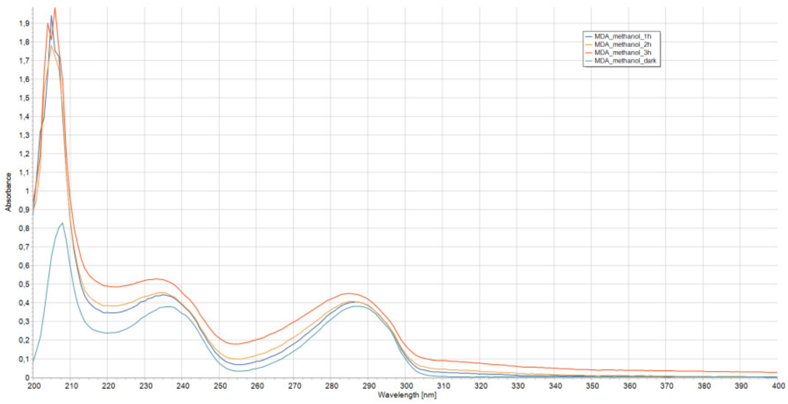

Figure S1C1

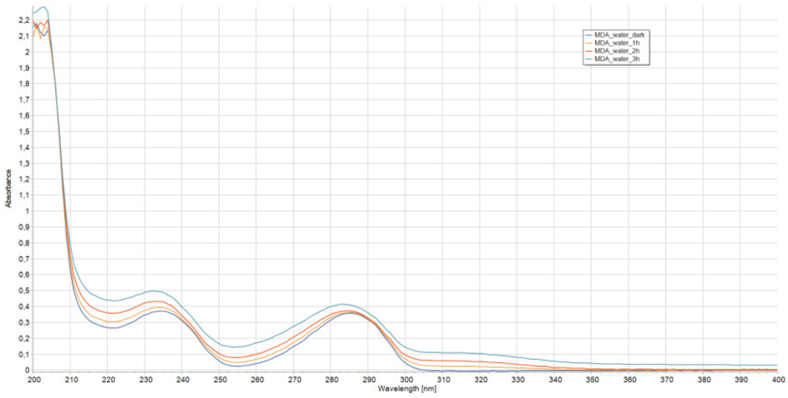

Figure S1C2

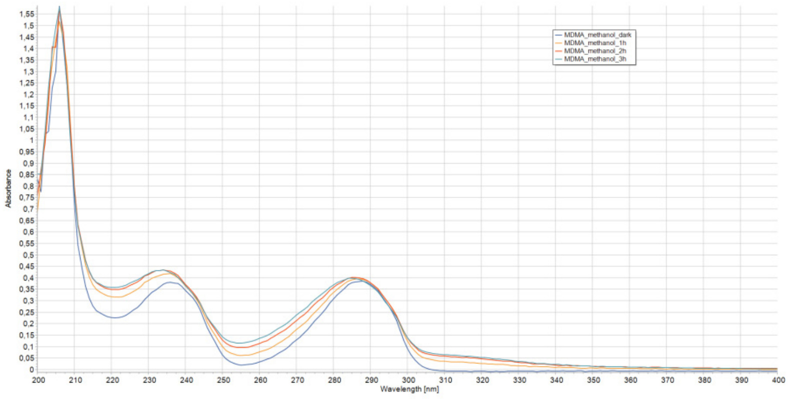

Figure S1D1

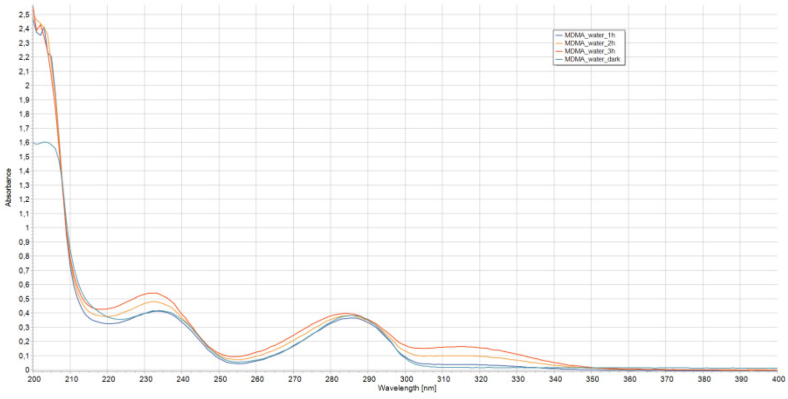

Figure S1D2

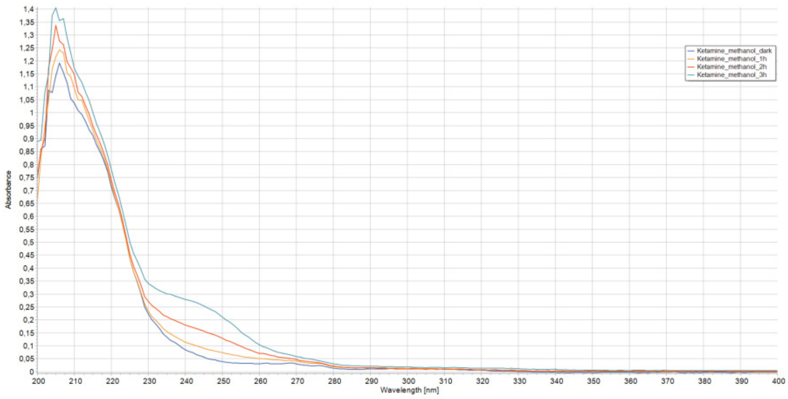

Figure S1E1

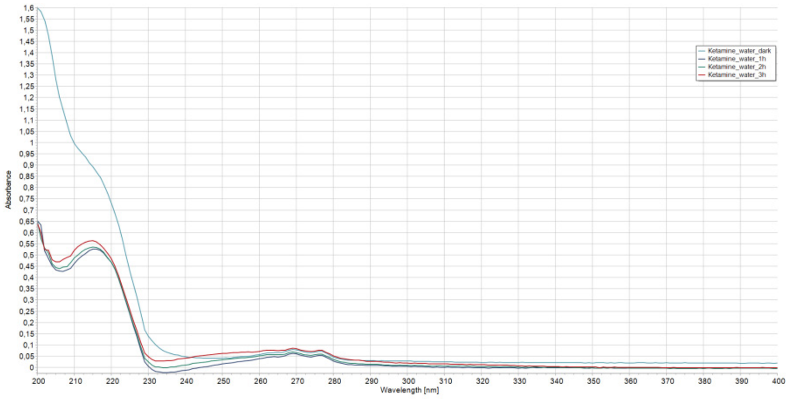

Figure S1E2

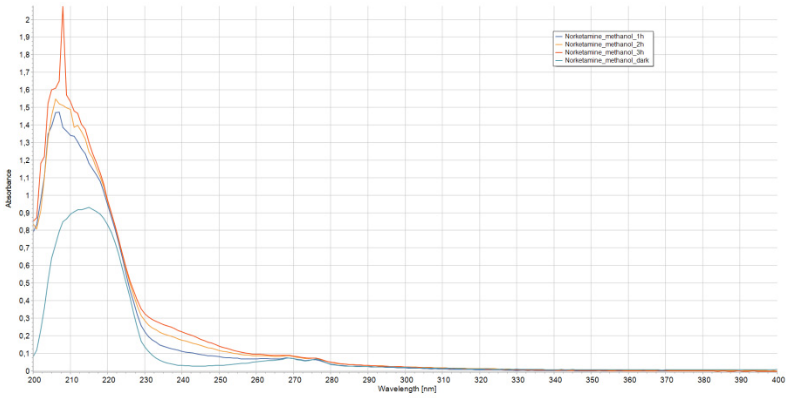

Figure S1F1

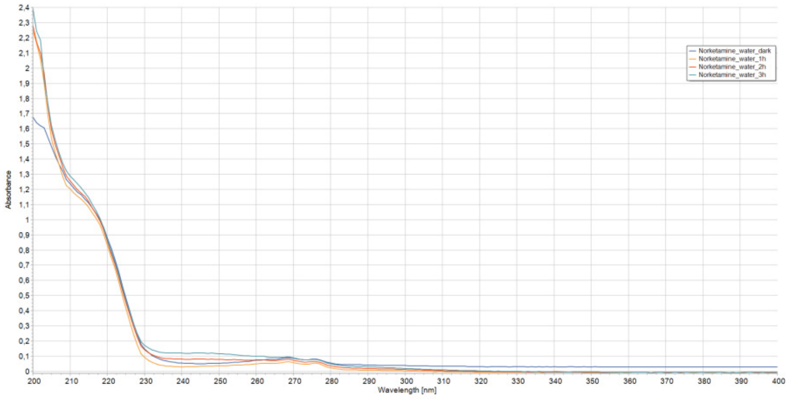

Figure S1F2

Captions for figures

Fig S1A1/A2. UV spectrum of AMF (10^{-4} M) in methanol/water, by increasing exposure time in solar simulator

Fig S1B1/B2. UV spectrum of MA (10^{-4} M) in methanol/water, by increasing exposure time in solar simulator

Fig S1C1/C2. UV spectrum of MDA (10^{-4} M) in methanol/water , by increasing exposure time in solar simulator

Fig S1D1/D2. UV spectrum of MDMA(10^{-4} M) in methanol/water, by increasing exposure time in solar simulator

Fig S1E1/E2. UV spectrum of KET (10^{-4} M) in methanol/water by increasing exposure time in solar simulator

Fig S1F1/F2. UV spectrum of NKET (10^{-4} M) in methanol/water, by increasing exposure time in solar simulator
